# Nasal-temporal asymmetric changes in retinal peripheral refractive error in myopic adolescents induced by overnight orthokeratology lenses

**DOI:** 10.3389/fneur.2022.1006112

**Published:** 2023-03-02

**Authors:** Xiaohang Chen, Yan Xiong, Xinzhou Qi, Longqian Liu

**Affiliations:** ^1^Laboratory of Optometry and Visual Science, Sichuan University, Chengdu, Sichuan, China; ^2^Department of Optometry and Visual Science, West China Hospital, Sichuan University, Chengdu, Sichuan, China; ^3^Department of Ophthalmology, West China Hospital, Sichuan University, Chengdu, Sichuan, China; ^4^Department of Pediatrics, Affiliated Hospital of North Sichuan Medical College, Nanchong, Sichuan, China; ^5^School of Management, Fudan University, Shanghai, China

**Keywords:** orthokeratology, myopia, peripheral refraction, relative peripheral refraction, axial length

## Abstract

**Objective:**

To observe the changes in peripheral refraction in myopic adolescents after overnight orthokeratology and its influencing factors.

**Methods:**

This was a prospective study among young myopic adolescents aged 8–14 years (*n* = 21). The peripheral refraction of the subjects was measured at 5, 10, 15, 20, 25, and 30° from the nasal and temporal side to the central fixation by WAM-5500 Open-field refractometer. The axial length, baseline spherical equivalent refraction, and other parameters were measured. The data were measured at baseline and 1, 3, and 12 months after wearing orthokeratology lenses.

**Results:**

The relative peripheral refraction at the nasal and temporal side from central to 30° eccentricity revealed relative hyperopic defocus in all subjects at baseline measurement. One month after wearing the orthokeratology lenses, the relative peripheral refraction changed to myopic defocus, the nasal-temporal relative peripheral refraction was asymmetric, and the observed difference was statistically significant. Positive correlations were found between the change amount of nasal relative peripheral refraction and baseline spherical equivalent refraction, the baseline nasal relative peripheral refraction was higher than that on the temporal side, and after orthokeratology, the value of nasal relative peripheral refraction was lower than that on the temporal side. The changes at 30° on both sides were correlated to the axial elongation (r_Nasal_ = 0.565, r_Temporal_ = 0.526, *p* < 0.05).

**Conclusion:**

This study demonstrated that after orthokeratology, relative peripheral hyperopia in the myopic patients turned into relative peripheral myopia, and the nasal-temporal asymmetry changed significantly after orthokeratology, which was correlated with the baseline refractive state.

## Introduction

Over the recent years, the prevalence of myopia has been steadily increasing worldwide (1, 2). Among all the available methods used to control myopia, orthokeratology has been receiving increasing attention as a safe and effective method (3–9).

While there are no definite conclusions regarding the principles and mechanisms of orthokeratology lens controlling myopia, according to previous studies, the comparative systematic and perfect possible mechanism might be explained by the theory of the peripheral refraction (10).

Peripheral Refraction (PR) is the retinal, refractive state of different eccentricities from the central fixation, and the relative peripheral refraction (RPR) is the refractive state of the peripheral retina relative to the central fixation (10). The PR can vary between myopia patients (11). The concept of peripheral refraction was first proposed by Hoogerheide and Rempt et al. (12, 13) in 1971. According to the classification of RPR, it can generally be divided into three types: relative peripheral hyperopia (RPH), relative peripheral myopia (RPM), and relative peripheral emmetropia (RPEm). Previous animal experiments have demonstrated that different peripheral defocus areas around the retina induce different refractive states (14–17).

The design of the orthokeratology lens is exactly in line with the optical principle of peripheral defocus (18). Previous studies have found that after wearing the orthokeratology lens, the peripheral refractive state of myopia patients goes through “myopic shift” (10, 19). It also shows the asymmetric change in the nasal and temporal side (20), where the asymmetry refraction might have more effect on the myopia progression.

The principle and mechanism of orthokeratology lens to control myopia have not been accurately determined. It is currently accepted that the reverse geometric design of the orthokeratology lens causes the RPM of the wearers. In the present study, we examined the influence of the orthokeratology lens on peripheral refraction and explored the nasal-temporal asymmetry changes of peripheral refraction after the orthokeratology.

## Materials and methods

### Study design

This prospectively designed, self-controlled observational study was conducted in accordance with the tenets of the Declaration of Helsinki and approval from the Ethics Committee of West China Hospital, Sichuan University (2017.43). This study was a part of our published study (21). All the subjects or their guardians signed written informed consent before being recruited into the study.

### Inclusion and exclusion criteria

This study included myopic patients aged 8~14 years, with the degree of myopia between – 1.00D and – 5.00D and the degree of astigmatism ≤ 1.50D. Good compliance and regular visits were also vital. Patients with contact lens contraindications (such as dry eye, eyelid gland dysfunction, eyelid insufficiency, allergic rhinitis, etc.), eye trauma surgery, severe allergies to cycloplegia drugs (compound toppicacamine eye drops) or contact lens care solution, strabismus, amblyopia, or other eye diseases, and general diseases (such as diabetes, Down's syndrome, rheumatoid arthritis, etc.) were excluded.

### Orthokeratology lens fitting process

Referring to the orthokeratology lens fitting standard (22), all subjects in this study were fitted with the same brand of orthokeratology lens so as to reduce the differences and effects between different brands and minimize such variations in different measurements due to various lens designs. The lenses were Euclid VST designs (Euclid Systems Corporation, USA). The lenses were manufactured with the oprifocon A (Boston Equalens II) which the oxygen permeability (DK) was 85 × 10^−11^ (cm^2^/s) [mLO_2_/(mL × mmHg)]. During the fitting process, the lens were ensured to maintain the contral position of the lens on the cornea, and the central postion of the overnight orthokeratology lens was checked by the corneal topography after the lens were removed by the wearers during the follow-up period.

### Peripheral refraction

The data from both eyes were highly correlated and only the data of the right eye were included in further analysis.

The PR of the right eye was measured by WAM-5500 open-field auto-refractor. To exclude the influence of accommodation in dynamic measurement, the patients were required to accept the cycloplegia (compound tropicamide eye drops) every 5 minutes for a total of 4 times. After the last time, they were required to close eyes and rest for 20 minutes, a penlight was used to evaluate the dilation of the pupil before the measurement, and the accommodative amplitude was measured to ensure the influence of accommodation have been eliminated.

**Figure 1:**
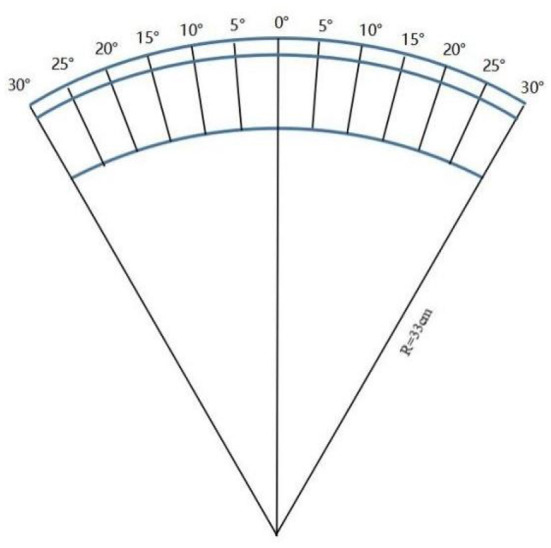
Arc measuring plate.

The subject, whose head was fixed by the frontal support and chin rest, were asked to stare at the measuring plate at 33 cm (Figure 1), the measuring plate was fixed to the rod, which is using to fix the visual target, after which the optometrist would move the Maltese cross along the scale (central fixation to nasal 30°, temporal 30°, 5° as dividing points), measuring each eccentricity for nine times and recording it with spherical equivalent (SE).

Relative Peripheral Refraction (RPR) is defined as the difference of refractive state between each eccentricity and the central retina. The RPR equation was as follows:


(1) $$\begin{array}{l} RPR=SE_{X}-SE_{c} \end{array}$$

where *SE*_*X*_ represents the SE of the X eccentricity from the center and *SE*_*c*_ represents the SE of the central fixation.

The initial spherical equivalent (SE_0_) (NIDEK RT-600, https://www.nidek-intl.com/), the intraocular pressure (IOP) (Canon TX-20, https://global.canon/en/) and axial length (AL) (ZEISSIOL Master 500, https://www.zeiss.com/meditec/int/home.html) were measured at BL, 1, 3, and 12 months.

### Statistical analysis

SPSS 22.0 and STATA16.0 software were used for the statistical analyses. Normality was tested with the Shapiro–Wilk normality test. Continuous variables were expressed as means with standard deviation (SD), or medians with the interquartile range (IQR), as appropriate. Single-factor repeated-measures analysis of variance (ANOVA) was used to compare the measured data at each time point. Paired *t*-test and Wilcoxon signed-rank test were used to observe the difference of RPR at each eccentricity. Non-parametric data were analyzed by Friedman test followed by Dunn's multiple comparisons test. Correlations were analyzed by using Spearman's (non-normality) or Pearson (normality) correlation. All values were rounded to three decimal digits. *P* < 0.05 represented a statistically significant difference.

## Results

### Demographics

A total of 21 subjects who accepted the peripheral refraction measurement were enrolled in the present study. They were measured at baseline (BL), 1, 3, and 12 months after wearing the lenses. The data from right eye were enrolled to RPR analysis to avoid bias. Among them, two subjects missed the last peripheral refraction measurement due to studying abroad and thus were excluded from the data processing. The demographics of the subjects are shown in Table 1.

**Table 1:** Clinical baseline data of the subjects.

	**Right eye**	**Left eye**
	**(*n* = 19)**	**(*n* = 19)**
Age(yr)	9.84 ± 1.64
**Sexuality**	
Male	10 (52.63%)
Female	9 (47.37%)
SE_0_(D)	– 2.73 ± 1.09	– 2.76 ± 1.19
IOP(mmHg)	15.82 ± 2.05	15.56 ± 2.52
AL(mm)	24.78 ± 0.93	24.80 ± 0.96

SE_0_, initial spherical equivalent; IOP, intraocular pressure; PD, pupil diameter; AL, axial length.

### Peripheral refraction

**Figure 2:**
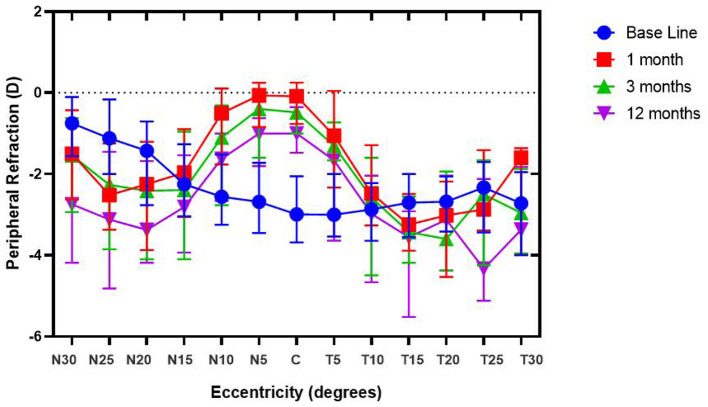
Peripheral refraction of subjects during the follow-up period. Relative peripheral refraction before (RPR_Baseline_) and after orthokeratology treatment (RPR_1/3/12month(s)_) for spherical equivalent (SE), with different degrees of eccentricity [30 nasal (N) to 30 temporal (T) across the retina].

**Figure 3:**
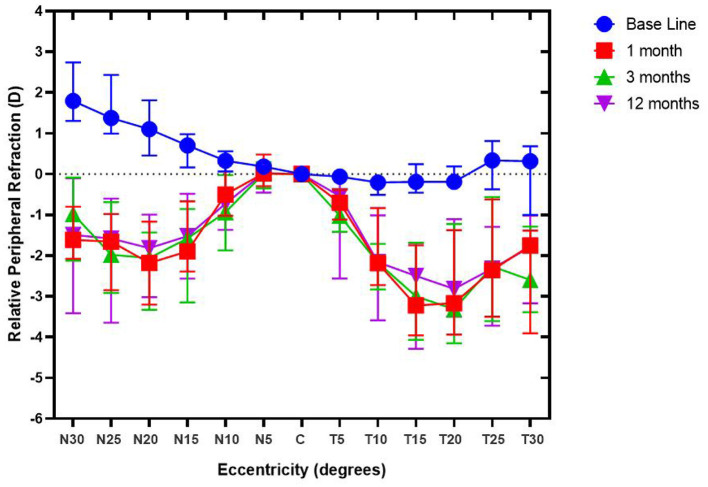
Relative peripheral refraction of subjects during the follow-up period. Relative peripheral refraction before (RPR_Baseline_) and after orthokeratology treatment (RPR_1/3/12month(s)_) for spherical equivalent (SE), with different degrees of eccentricity [30 nasal (N) to 30 temporal (T) across the retina].

The PR of these subjects was measured before and after the orthokeratology lens (Figure 2). Also, the RPR of subjects is shown in Figure 3. During the follow-up period, the defocus area around the nasal and temporal periphery changed. Figure 3 shows the RPR changes from relative hyperopic to relative myopic defocus. The significant differences of RPR at each eccentricity in the different follow-up periods were calculated (Table 2).

**Table 2:** Relative peripheral refraction (Mean ± SD) after treatment for each eccentric nasal (N) and temporal (T) side; statistical significance for comparison between pre- and post-treatment (p^a^), and significance for the comparison of relative peripheral refraction at corresponding nasal-temporal side among each follow-up record (p^b^).

**Eccentricity**	**RPR(p^a^)**	**Eccentricity**	**RPR(p^a^)**	**[p^b^]**
**N30**		**T30**		
Base Line	2.197 ± 1.128	Base Line	– 0.033 ± 1.177	< 0.0001*
1 month	– 1.366 ± 1.267( < 0.0001*)	1 month	– 2.048 ± 1.789( < 0.0001*)	0.057*
3 months	– 0.949 ± 2.001(** < 0.0001+**)	3 months	– 2.571 ± 1.568( < 0.0001*)	**0.024+**
12 months	– 1.722 ± 1.758(** < 0.0001+**)	12 months	– 2.081 ± 1.748( < 0.0001*)	0.260+
**N25**		**T25**		
Base Line	1.673 ± 0.898	Base Line	0.278 ± 0.800	< 0.0001*
1 month	– 1.796 ± 1.372( < 0.0001*)	1 month	– 1.959 ± 1.763( < 0.0001*)	0.646*
3 months	– 1.873 ± 1.411( < 0.0001*)	3 months	– 2.135 ± 2.094( < 0.0001*)	0.534*
12 months	– 2.101 ± 1.781( < 0.0001*)	12 months	– 2.621 ± 1.651( < 0.0001*)	0.276*
**N20**		**T20**		
Base Line	1.121 ± 0.732	Base Line	– 0.054 ± 0.701	< 0.0001*
1 month	– 2.054 ± 1.493( < 0.0001*)	1 month	– 2.984 ± 1.788( < 0.0001*)	0.001*
3 months	– 2.214 ± 1.194( < 0.0001*)	3 months	– 2.894 ± 1.875( < 0.0001*)	0.071*
12 months	– 2.101 ± 1.725( < 0.0001*)	12 months	– 2.407 ± 1.833( < 0.0001*)	0.442*
**N15**		**T15**		
Base Line	0.652 ± 0.484	Base Line	– 0.130 ± 0.600	< 0.0001*
1 month	– 1.466 ± 1.283( < 0.0001*)	1 month	– 2.857 ± 1.560( < 0.0001*)	0.001*
3 months	– 1.877 ± 1.321( < 0.0001*)	3 months	– 3.011 ± 1.489( < 0.0001*)	0.009*
12 months	– 1.646 ± 1.501( < 0.0001*)	12 months	– 2.999 ± 1.853(< 0.0001*)	0.006*
**N10**		**T10**		
Base Line	0.327 ± 0.369	Base Line	– 0.205 ± 0.433	< 0.0001*
1 month	– 0.586 ± 1.037(0.001*)	1 month	– 2.268 ± 1.534(0.0001*)	0.001*
3 months	– 0.896 ± 1.289(0.002*)	3 months	– 2.356 ± 1.475(< 0.0001*)	0.002*
12 months	– 0.777 ± 1.099(0.001*)	12 months	– 2.382 ± 1.615(< 0.0001*)	< 0.0001*
**N5**		**T5**		
Base Line	0.207 ± 0.282	Base Line	– 0.027 ± 0.391	**0.009+**
1 month	– 0.053 ± 0.842(0.409+)	1 month	– 0.871 ± 0.817(0.002*)	0.009*
3 months	– 0.137 ± 0.687(0.091+)	3 months	– 0.958 ± 0.634(0.0002*)	0.001*
12 months	– 0.169 ± 0.648(**0.027+**)	12 months	– 1.147 ± 1.305(** < 0.0001+**)	**0.018+**

(^*^) parametric tests; (+) Non-parametric tests; boldface: *p* < 0.05.

**Figure 4:**
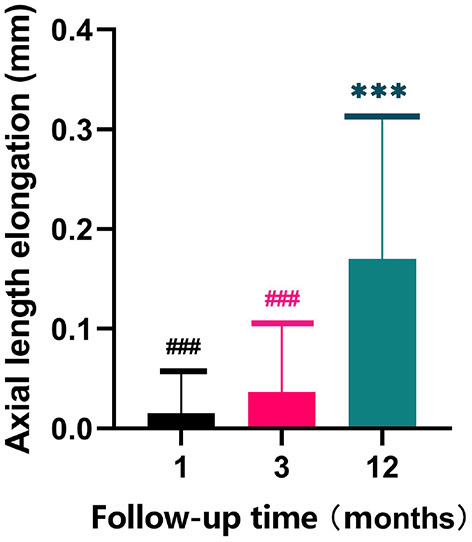
Axial length elongation of subjects during the follow-up period. ***represent the significance between the values at the 12th month follow-up and baseline (*p* < 0.001); ^**###**^ represent the significance between the values at the 1st, 3rd and 12th month follow-up (*p* < 0.001).

During the follow-up period, the mean value of the axial length in right eyes of the participants was 24.78 ± 0.93 mm, the mean value of the axial length of subjects after 12 months follow-up period was 24.95 ± 0.88 mm. The mean value of axial length elongation was 0.01 ± 0.04 mm at the 1st month follow-up, 0.04 ± 0.07 mm at the 3rd month follow-up and 0.17 ± 0.14 mm at the 12th month follow-up (Figure 4), it suggested that the the value at the end of the follow-up (12th month follow-up) was significantly different from the baseline value (*p* = 0.0004).

Table 2 demonstrated that the nasal-temporal asymmetry still existed before and after the orthokeratology lens, however, it should be noted that the difference changed after the orthokeratology lens. The value of nasal RPR at each eccentricity was larger than that in the temporal RPR before orthokeratology. In contrast, after the orthokeratology lens, the value of the temporal RPR at different eccentricities was larger than that of the nasal RPR. However, the asymmetry of RPR in the nasal and temporal side existed until 20° eccentricity after orthokeratology, while beyond the 20–30° of each eccentricity, it showed fewer asymmetric changes.

**Figure 5:**
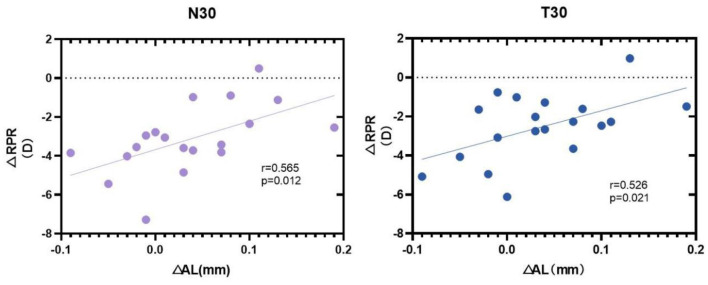
Correlation analyses of change amount between change amount of axial length (ΔAL) and the change amount of relative peripheral refraction (ΔRPR) at nasal 30° (N30), Temporal 30° (T30) after 3 months of orthokeratology lens wearing.

After 3 months, the change amount of axial length (ΔAL) was associated with the change amount of relative peripheral refraction (ΔRPR) at the N30 (*p* = 0.012), T5 (*p* = 0.042), T10 (*p* = 0.014), and T30 (*p* = 0.021), and there were no significant changes in other positions (Figure 5).


(2) $$\begin{array}{l} \Delta \text{RPR}={\text{RPR}}_{3}-{\text{RPR}}_{0} \end{array}$$

(3) $$\begin{array}{l} \Delta \text{AL}={\text{AL}}_{3}-{\text{AL}}_{0} \end{array}$$

After 1 month of orthokeratology, the change amount of ΔRPR was not related to the SE_0_ (Table 3). After 3 months of wearing the lens, a positive correlation was found between the ΔRPR and the SE_0_, and the asymmetric effect on the nasal and temporal side was observed. Also, a stronger correlation was found on the nasal side. One year after wearing the lens, the number of ΔRPR changes associated with the SE_0_ decreased at different eccentricities but still showed nasal and temporal asymmetry and a stronger correlation with the number of nasal RPR changes.

**Table 3:** Correlation analyses between the RPR change amount and SE_0_ during the follow-up period (Controlling for age and sex).

**Follow-up period (months)**	**N30**	**N25**	**N20**	**N15**	**N10**	**N5**	**T5**	**T10**	**T15**	**T20**	**T25**	**T30**
1												
r	0.472	0.485	0.338	0.066	– 0.027	– 0.044	0.587	0.486	0.142	0.337	0.545	0.603
p	0.056	0.049	0.185	0.803	0.919	0.866	0.013	0.048	0.586	0.186	0.024	0.010
3												
r	0.625	0.643	0.556	0.428	0.626	0.317	0.449	0.511	0.399	0.468	0.407	0.539
p	0.007	0.005	0.020	0.087	0.007	0.215	0.071	0.036	0.112	0.058	0.105	0.026
12												
r	0.286	0.479	0.181	0.414	0.011	– 0.439	0.228	0.408	0.235	0.273	0.447	0.208
p	0.266	0.052	0.487	0.099	0.967	0.078	0.378	0.104	0.364	0.288	0.072	0.424

## Discussion

Due to the unique design of the orthokeratology lens, when the lens covers the wearer's cornea, the reverse geometric design of the lens can come in direct contact with the cornea's front surface, thus changing the corneal curvature (19, 23) and RPR through some mechanisms (10). In addition to reshaping the corneal form, the orthokeratology lens may function under the principle of peripheral refraction to control myopia. This study measured the orthokeratology lens wearer in the nasal, temporal gaze range of the refractive state. Our results revealed that the RPR of myopia patients changed after orthokeratology. We also highlighted the asymmetric changes of RPR at the nasal and temporal side before and after the orthokeratology lens and explored the reasons that might cause the asymmetric RPR.

Since horizontal PR has been more significantly related to myopia than vertical PR (24, 25), only horizontal PR was measured in this study. Most subjects were RPH before the orthokeratology. Preclinical studies showed that peripheral retinal focus could promote myopia progression (26, 27). In their clinical studies, Hoogerheide and Rempt et al. (12, 13) firstly suggested that the myopia progression in patients with RPH was faster than in those with RPM in the same age group, while the young adults with positive parental myopia had increasing AL and RPH compared to the control group with negative parental myopia (28).

This study found that after the orthokeratology, the RPR of wearers changed from PRH to PRM, and the difference was statistically significant. These results prove that the design principle of the orthokeratology lens does affect the RPR of the lens wearers (29). The previous study compared the RPR of myopic patients with a single vision frame lens, revealing that the subjects with a single vision frame lens did not change with PRH, and the orthokeratology group patients changed from PRH to PRM, which was consistent with our study results (30, 31). According to the results of this study, the initial nasal RPR was larger than that on the temporal side. After wearing the orthokeratology lens, the value of nasal RPR was smaller than that in the temporal side and changed into PRM. Moreover, it seems that orthokeratology promoted the symmetric RPR changes in the nasal and temporal side of lens wearers (19). The specific cause of the RPR changes might be related to the mechanism of the myopia control effect exerted by the orthokeratology lens.

This study also found that the RPR amount of N30 and T30 after 3 months of lens wearing was positively correlated to the AL. This conclusion is similar to the results of Gifford et al. (30, 31), who confirmed that the peripheral defocus could directly affect the number of changes in the AL, i.e., the progression of myopia (32, 33) suggested that the change of the AL could be preliminarily predicted according to the change of RPR.

Based on the results of the one-year follow-up, the researchers found that the RPR at the nasal side was positively related to the SE_0_, which is similar to the results of Charman et al. (34), and might be one of the reasons explaining the asymmetric changes in the nasal and temporal side. These results can serve as a reference for designing individualized orthokeratology lenses for better myopia control efficacy.

Nevertheless, this study had some shortcomings, the small sample size leading to bias in the results, so further research on the issue should be conducted with a larger sample size. Furthermore, our study was limited in terms of follow-up time, and follow-up duration of this study need to be improved as well.

## Conclusion

Taken together, these results suggested that the correlations mentioned below revealed a nasal-temporal asymmetry in RPR, where the SE_0_ was more strongly correlated with the nasal RPR changes. The correlation was one of the reasons for the asymmetric changes in the nasal, temporal RPR after orthokeratology, which can serve as a reference for designing individualized orthokeratology lenses for better myopia control efficacy.

## Data availability statement

The raw data supporting the conclusions of this article will be made available by the authors, without undue reservation.

## Ethics statement

The studies involving human participants were reviewed and approved by Ethics Committee of West China Hospital, Sichuan University. Written informed consent to participate in this study was provided by the participants' legal guardian/next of kin.

## Author contributions

Study design, concept, and data extracting: XC, YX, and LL. Database search: XC and YX. Data analysis: XC and XQ. Manuscript writing: XC. Manuscript revising: XC and LL. All authors approved the final version of the manuscript.

## References

[1] Sankaridurg P Tahhan N Kandel H Naduvilath T Zou H Frick KD. IMI impact of myopia. Invest Ophthalmol Vis Sci. (2021) 62:2. 10.1167/iovs.62.5.2 33909036 PMC8083082

[2] Wang J Li Y Musch DC Wei N Qi X Ding G. Progression of myopia in school-aged children After COVID-19 home confinement. JAMA Ophthalmol. (2021) 139:293. 10.1001/jamaophthalmol.2020.6239 33443542 PMC7809617

[3] Hiraoka T Kakita T Okamoto F Takahashi H Oshika T. Long-term effect of overnight orthokeratology on axial length elongation in childhood myopia: a 5-year follow-up study. Invest Ophthalmol Vis Sci. (2012) 53:3913–9. 10.1167/iovs.11-8453 22577080

[4] Santodomingo-Rubido J Villa-Collar C Gilmartin B Gutierrez-Ortega R. Myopia control with orthokeratology contact lenses in Spain: refractive and biometric changes. Invest Ophthalmol Vis Sci. (2012) 53:5060–5. 10.1167/iovs.11-8005 22729437

[5] Cho P Cheung SW. Retardation of myopia in Orthokeratology (ROMIO) study: a 2-year randomized clinical trial. Invest Ophthalmol Vis Sci. (2012) 53:7077–85. 10.1167/iovs.12-10565 22969068

[6] Santodomingo-Rubido J Villa-Collar C Gilmartin B Gutierrez-Ortega R Sugimoto K. Long-term efficacy of orthokeratology contact lens wear in controlling the progression of childhood myopia. Curr Eye Res. (2017) 42:713–20. 10.1080/02713683.2016.1221979 27767354

[7] Wildsoet CF Chia A Cho P Guggenheim JA Polling JR Read S. IMI—interventions myopia institute: interventions for controlling myopia onset and progression report. Invest Ophthalmol Vis Sci. (2019) 60:M106–31. 10.1167/iovs.18-25958 30817829

[8] Bullimore MA Johnson LA. Overnight orthokeratology. Cont Lens Anterior Eye. (2020) 43:322–32. 10.1016/j.clae.2020.03.018 32331970

[9] Lee JH Hong IH Lee TY Han JR Jeon GS. Choroidal thickness changes after orthokeratology lens wearing in young adults with myopia. Ophthalmic Res. (2021) 64:121–7. 10.1159/000510715 32759609

[10] Gifford KL Gifford P Hendicott PL Schmid KL. Stability of peripheral refraction changes in orthokeratology for myopia. Cont Lens Anterior Eye. (2020) 43:44–53. 10.1016/j.clae.2019.11.008 31796369

[11] Ding X Lin Z Huang Q Zheng Y Congdon N He M. Heritability of peripheral refraction in Chinese children and adolescents: the Guangzhou Twin Eye study. Invest Ophth Vis Sci. (2012) 53:107–11. 10.1167/iovs.11-8716 22159008

[12] Hoogerheide J Rempt F Hoogenboom WP. Acquired myopia in young pilots. Ophthalmologica. (1971) 163:209–15. 10.1159/000306646 5127164

[13] Rempt F Hoogerheide J Hoogenboom WP. Peripheral retinoscopy and the skiagram. Ophthalmologica. (1971) 162:1–10. 10.1159/000306229 5547863

[14] Benavente-Perez A Nour A Troilo D. The effect of simultaneous negative and positive defocus on eye growth and development of refractive state in marmosets. Invest Ophthalmol Vis Sci. (2012) 53:6479–87. 10.1167/iovs.12-9822 22918633 PMC3450918

[15] Liu Y Wildsoet C. The effective add inherent in 2-zone negative lenses inhibits eye growth in myopic young chicks. Invest Ophthalmol Vis Sci. (2012) 53:5085–93. 10.1167/iovs.12-9628 22761258 PMC3410667

[16] Benavente-Perez A Nour A Troilo D. Axial eye growth and refractive error development can be modified by exposing the peripheral retina to relative myopic or hyperopic defocus. Invest Ophthalmol Vis Sci. (2014) 55:6765–73. 10.1167/iovs.14-14524 25190657 PMC4209715

[17] Troilo D Smith REL Nickla DL Ashby R Tkatchenko AV Ostrin LA. IMI—report on experimental models of emmetropization and myopia. Invest Ophth Vis Sci. (2019) 60:M31–88. 10.1167/iovs.18-25967 30817827 PMC6738517

[18] Lee TT Cho P. Repeatability of relative peripheral refraction in untreated and orthokeratology-treated eyes. Optom Vis Sci. (2012) 89:1477–86. 10.1097/OPX.0b013e31826912cd 22940780

[19] Gonzalez-Meijome JM Faria-Ribeiro MA Lopes-Ferreira DP Fernandes P Carracedo G Queiros A. Changes in peripheral refractive profile after orthokeratology for different degrees of myopia. Curr Eye Res. (2016) 41:199–207. 10.3109/02713683.2015.1009634 25803198

[20] Queiros A Gonzalez-Meijome JM Jorge J Villa-Collar C Gutierrez AR. Peripheral refraction in myopic patients after orthokeratology. Optom Vis Sci. (2010) 87:323–9. 10.1097/OPX.0b013e3181d951f7 20375751

[21] Chen X Xiong Y Liu F Wang J Yang B Liu L. Factors determining the myopia control effect of an orthokeratology lens: a two-year multi-level model. Ophthal Physl Opt. (2022) 42:786–96. 10.1111/opo.12990 35499112

[22] Vincent SJ Cho P Chan KY Fadel D Ghorbani-Mojarrad N González-Méijome JM. CLEAR—orthokeratology. Contact Lens Anterior Eye. (2021) 44:240–69. 10.1016/j.clae.2021.02.003 33775379

[23] Nieto-Bona A Gonzalez-Mesa A Nieto-Bona MP Villa-Collar C Lorente-Velazquez A. Long-term changes in corneal morphology induced by overnight orthokeratology. Curr Eye Res. (2011) 36:895–904. 10.3109/02713683.2011.593723 21950694

[24] Atchison DA Pritchard N Schmid KL. Peripheral refraction along the horizontal and vertical visual fields in myopia. Vision Res. (2006) 46:1450–8. 10.1016/j.visres.2005.10.023 16356528

[25] Mutti DO Sinnott LT Reuter KS Walker MK Berntsen DA Jones-Jordan LA. Peripheral refraction and eye lengths in myopic children in the bifocal lenses in nearsighted kids (BLINK) study. Transl Vis Sci Techn. (2019) 8:17. 10.1167/tvst.8.2.17 31019848 PMC6469879

[26] Smith ER Huang J Hung LF Blasdel TL Humbird TL Bockhorst KH. Hemiretinal form deprivation: evidence for local control of eye growth and refractive development in infant monkeys. Invest Ophthalmol Vis Sci. (2009) 50:5057–69. 10.1167/iovs.08-3232 19494197 PMC2778320

[27] Wallman J Gottlieb M Rajaram V Fugate-Wentzek L. Local retinal regions control local eye growth and myopia. Science. (1987) 237:73–7. 10.1126/science.3603011 3603011

[28] Koomson NY Kobia-Acquah E Abdul-Kabir M Aderonke UM Kwaw RJ Arkhurst EE. Relationship between peripheral refraction, axial lengths and parental myopia of young adult myopes. J Optom. (2021). 10.1016/j.optom.2020.10.007 33531294 PMC9068532

[29] Swarbrick HA Alharbi A Watt K Lum E Kang P. Myopia control during orthokeratology lens wear in children using a novel study design. Ophthalmology. (2015) 122:620–30. 10.1016/j.ophtha.2014.09.028 25439432

[30] Lin Z Martinez A Chen X Li L Sankaridurg P Holden BA. Peripheral defocus with single-vision spectacle lenses in myopic children. Optom Vis Sci. (2010) 87:4–9. 10.1097/OPX.0b013e3181c078f1 19826316

[31] Backhouse S Fox S Ibrahim B Phillips JR. Peripheral refraction in myopia corrected with spectacles vs. contact lenses. Ophthalmic Physiol Opt. (2012) 32:294–303. 10.1111/j.1475-1313.2012.00912.x 22577970

[32] Kim J Lim DH Han SH Chung T. Predictive factors associated with axial length growth and myopia progression in orthokeratology. PLoS ONE. (2019) 14:e218140. 10.1371/journal.pone.0218140 31188890 PMC6561598

[33] Khorrami-Nejad M Moradi R Akbarzadeh BA Khosravi B. Effect of axial length and anterior chamber depth on the peripheral refraction profile. Int J Ophthalmol. (2021) 14:292–8. 10.18240/ijo.2021.02.17 33614460 PMC7840374

[34] Charman WN Mountford J Atchison DA Markwell EL. Peripheral refraction in orthokeratology patients. Optom Vis Sci. (2006) 83:641–8. 10.1097/01.opx.0000232840.66716.af 16971842

